# Supplementary material for: Resolving the full spectrum of human genome variation using Linked-Reads
Source: Genome Res. 2019 Apr;29(4):635–45. doi: 10.1101/gr.234443.118 (PMC6442396; doi:10.1101/gr.234443.118)
Supplement: Supplemental Material [file supp_gr.234443.118_Supplemental_longranger-2.2.zip › longranger-2.2.tar.bz2!/lr2.2-public/lib/stan/cmdstan/stan/doc/api/html/list__argument_8hpp_source.html]

Stan: src/stan/gm/arguments/list\_argument.hpp Source File


|  |  |
| --- | --- |
|  | Stan  2.5.0  probability, sampling & optimization |


- Main Page
- Related Pages
- Namespaces
- Classes
- Files

- File List
- File Members

All Classes Namespaces Files Functions Variables Typedefs Enumerations Enumerator Friends Macros Pages

- src
- stan
- gm
- arguments

list\_argument.hpp

Go to the documentation of this file.

1 #ifndef STAN\_\_GM\_\_ARGUMENTS\_\_LIST\_\_ARGUMENT\_\_BETA

2 #define STAN\_\_GM\_\_ARGUMENTS\_\_LIST\_\_ARGUMENT\_\_BETA

3

4 #include <iostream>

5 #include <stan/gm/arguments/valued\_argument.hpp>

6 #include <stan/gm/arguments/arg\_fail.hpp>

7

8 namespace stan {

9

10  namespace gm {

11

12  class list\_argument: public valued\_argument {

13

14  public:

15

16  list\_argument() {

17  \_value\_type = "list element";

18  }

19

20  ~list\_argument() {

21

22  for (std::vector<argument\*>::iterator it = \_values.begin();

23  it != \_values.end(); ++it) {

24  delete \*it;

25  }

26

27  \_values.clear();

28

29  }

30

31  void print(std::ostream\* s, int depth, const std::string prefix) {

32  valued\_argument::print(s, depth, prefix);

33  \_values.at(\_cursor)->print(s, depth + 1, prefix);

34  }

35

36  void print\_help(std::ostream\* s, int depth, bool recurse) {

37  \_default = \_values.at(\_default\_cursor)->name();

38

39  valued\_argument::print\_help(s, depth);

40

41  if (recurse) {

42  for (std::vector<argument\*>::iterator it = \_values.begin();

43  it != \_values.end(); ++it)

44  (\*it)->print\_help(s, depth + 1, true);

45  }

46  }

47

48  bool parse\_args(std::vector<std::string>& args, std::ostream\* out,

49  std::ostream\* err, bool& help\_flag) {

50

51  if(args.size() == 0) return true;

52

53  std::string name;

54  std::string value;

55  split\_arg(args.back(), name, value);

56

57  if(\_name == "help") {

58  print\_help(out, 0, false);

59  help\_flag |= true;

60  args.clear();

61  return false;

62  }

63  else if(\_name == "help-all") {

64  print\_help(out, 0, true);

65  help\_flag |= true;

66  args.clear();

67  return false;

68  }

69  else if(\_name == name) {

70

71  args.pop\_back();

72

73  bool good\_arg = false;

74  bool valid\_arg = true;

75

76  for (size\_t i = 0; i < \_values.size(); ++i) {

77  if( \_values.at(i)->name() != value) continue;

78

79  \_cursor = i;

80  valid\_arg &= \_values.at(\_cursor)->parse\_args(args, out, err, help\_flag);

81  good\_arg = true;

82  break;

83  }

84

85  if(!good\_arg) {

86

87  if(err) {

88  \*err << value << " is not a valid value for \"" << \_name << "\"" << std::endl;

89  \*err << std::string(indent\_width, ' ') << "Valid values:" << print\_valid() << std::endl;

90  }

91

92  args.clear();

93  }

94

95  return valid\_arg && good\_arg;

96

97  }

98

99  return true;

100

101  };

102

103  virtual void probe\_args(argument\* base\_arg, std::stringstream& s) {

104

105  for (size\_t i = 0; i < \_values.size(); ++i) {

106  \_cursor = i;

107

108  s << "good" << std::endl;

109  base\_arg->print(&s, 0, "");

110  s << std::endl;

111

112  \_values.at(i)->probe\_args(base\_arg, s);

113  }

114

115  \_values.push\_back(new arg\_fail);

116  \_cursor = \_values.size() - 1;

117  s << "bad" << std::endl;

118  base\_arg->print(&s, 0, "");

119  s << std::endl;

120

121  \_values.pop\_back();

122  \_cursor = \_default\_cursor;

123

124  }

125

126  void find\_arg(std::string name,

127  std::string prefix,

128  std::vector<std::string>& valid\_paths) {

129

130  if (name == \_name) {

131  valid\_paths.push\_back(prefix + \_name + "=<list\_element>");

132  }

133

134  prefix += \_name + "=";

135  for (std::vector<argument\*>::iterator it = \_values.begin();

136  it != \_values.end(); ++it) {

137  std::string value\_prefix = prefix + (\*it)->name() + " ";

138  (\*it)->find\_arg(name, prefix, valid\_paths);

139  }

140

141  }

142

143  bool valid\_value(std::string name) {

144  for (std::vector<argument\*>::iterator it = \_values.begin();

145  it != \_values.end(); ++it)

146  if (name == (\*it)->name())

147  return true;

148  return false;

149  }

150

151  argument\* arg(std::string name) {

152  if(name == \_values.at(\_cursor)->name())

153  return \_values.at(\_cursor);

154  else

155  return 0;

156  }

157

158  std::vector<argument\*>& values() { return \_values; }

159

160  std::string value() { return \_values.at(\_cursor)->name(); }

161

162  std::string print\_value() { return \_values.at(\_cursor)->name(); }

163

164  std::string print\_valid() {

165  std::string valid\_values;

166

167  std::vector<argument\*>::iterator it = \_values.begin();

168  valid\_values += " " + (\*it)->name();

169  ++it;

170

171  for (; it != \_values.end(); ++it)

172  valid\_values += ", " + (\*it)->name();

173

174  return valid\_values;

175

176  }

177

178  bool is\_default() { return \_cursor == \_default\_cursor; }

179

180  protected:

181

182  int \_cursor;

183  int \_default\_cursor;

184

185  std::vector<argument\*> \_values;

186

187  };

188

189  } // gm

190

191 } // stan

192

193 #endif

stan::gm::list\_argument::print\_value

std::string print\_value()

**Definition:** list\_argument.hpp:162

arg\_fail.hpp

stan::gm::valued\_argument::print\_help

virtual void print\_help(std::ostream \*s, const int depth, const bool recurse=false)

**Definition:** valued\_argument.hpp:26

stan::gm::list\_argument::arg

argument \* arg(std::string name)

**Definition:** list\_argument.hpp:151

stan::gm::list\_argument::print\_valid

std::string print\_valid()

**Definition:** list\_argument.hpp:164

stan::gm::list\_argument::\_default\_cursor

int \_default\_cursor

**Definition:** list\_argument.hpp:183

stan::gm::list\_argument::find\_arg

void find\_arg(std::string name, std::string prefix, std::vector< std::string > &valid\_paths)

**Definition:** list\_argument.hpp:126

stan::gm::valued\_argument::\_default

std::string \_default

**Definition:** valued\_argument.hpp:47

stan::gm::list\_argument::\_values

std::vector< argument \* > \_values

**Definition:** list\_argument.hpp:185

stan::gm::list\_argument

**Definition:** list\_argument.hpp:12

stan::gm::argument::split\_arg

static void split\_arg(const std::string &arg, std::string &name, std::string &value)

**Definition:** argument.hpp:54

valued\_argument.hpp

stan::gm::valued\_argument

**Definition:** valued\_argument.hpp:10

stan::gm::list\_argument::~list\_argument

~list\_argument()

**Definition:** list\_argument.hpp:20

stan::gm::valued\_argument::\_value\_type

std::string \_value\_type

**Definition:** valued\_argument.hpp:48

stan::gm::list\_argument::probe\_args

virtual void probe\_args(argument \*base\_arg, std::stringstream &s)

**Definition:** list\_argument.hpp:103

stan::gm::list\_argument::print\_help

void print\_help(std::ostream \*s, int depth, bool recurse)

**Definition:** list\_argument.hpp:36

stan::gm::argument::\_name

std::string \_name

**Definition:** argument.hpp:76

stan::gm::argument::indent\_width

int indent\_width

**Definition:** argument.hpp:79

stan::gm::valued\_argument::print

virtual void print(std::ostream \*s, const int depth, const std::string prefix)

**Definition:** valued\_argument.hpp:14

stan::gm::arg\_fail

**Definition:** arg\_fail.hpp:10

stan::gm::list\_argument::\_cursor

int \_cursor

**Definition:** list\_argument.hpp:182

stan::gm::list\_argument::parse\_args

bool parse\_args(std::vector< std::string > &args, std::ostream \*out, std::ostream \*err, bool &help\_flag)

**Definition:** list\_argument.hpp:48

stan::gm::list\_argument::is\_default

bool is\_default()

**Definition:** list\_argument.hpp:178

stan::gm::list\_argument::valid\_value

bool valid\_value(std::string name)

**Definition:** list\_argument.hpp:143

stan::gm::argument::name

std::string name() const

**Definition:** argument.hpp:26

stan::gm::argument::print

virtual void print(std::ostream \*s, const int depth, const std::string prefix)=0

stan::gm::list\_argument::values

std::vector< argument \* > & values()

**Definition:** list\_argument.hpp:158

stan::gm::argument

**Definition:** argument.hpp:12

stan::gm::list\_argument::value

std::string value()

**Definition:** list\_argument.hpp:160

stan::gm::list\_argument::print

void print(std::ostream \*s, int depth, const std::string prefix)

**Definition:** list\_argument.hpp:31

stan::gm::list\_argument::list\_argument

list\_argument()

**Definition:** list\_argument.hpp:16


---

[ Stan Home Page ]

*© 2011–2014,
Stan Development Team.*
